# Quantitative proteomic analysis of Bi Zhong Xiao decoction against collagen-induced arthritis rats in the early and late stages

**DOI:** 10.1186/s12906-022-03663-5

**Published:** 2022-07-13

**Authors:** Cailin He, Yang Wang, Yuqi Wen, Teng Li, En Hu, Siqing Zeng, Xingui Xiong

**Affiliations:** 1 Department of Integrated Traditional Chinese and Western Medicine, Institute of Integrative Medicine, Xiangya Hospital, Central South University, Changsha, 410008 China; 2 National Clinical Research Center for Geriatric Disorders, Xiangya Hospital, Central South University, Changsha, Hunan P.R. China 410008

**Keywords:** Bi Zhong Xiao decoction, Collagen-induced arthritis, Proteomics, Rheumatoid arthritis, Traditional Chinese medicine

## Abstract

**Background:**

Rheumatoid arthritis (RA) is a chronic, progressive, systemic autoimmune inflammatory disease. Bi Zhong Xiao decoction (BZXD) performs multiple functions for rheumatoid arthritis (RA) treatment for decades. In this study, we aimed to study the protein alterations of BZXD in the early and late stages of RA.

**Methods:**

Sprague–Dawley rats were randomly divided into the Control, collagen-induced arthritis (CIA) and BZXD groups. Clinical assessment, paw thickness, weight changes and serum inflammatory cytokine levels were used to evaluate anti-inflammatory effects. Histopathological tests were performed to assess the improvement of inflammation and synovial hyperplasia. Moreover, we analyzed the proteins profiling of synovial tissue samples with different time intervals after BZXD treatment by Isobaric Tag for Relative Absolute (ITRAQ) quantitative proteomics technology. To further explore the interrelationships among differentially expressed proteins (DEPs), we used DAVID Bioinformatics Resources v6.8 and STRING 11.0 for bioinformatics analysis. Besides, the western blot and immunohistochemistry were exerted to verify related proteins.

**Results:**

In our study, BZXD ameliorated joint inflammation, and suppressed the pathological changes in arthrosis of CIA rats. The proteomic analysis demonstrated that CIA rats were mainly involved in two significant pathways (the focal adhesion and the ECM-receptor interaction) in the early stage. BZXD down-regulated the expression of proteins involved in these pathways, such as CAV1, CHAD, COL3A1, COL5A2, COL6A1, and COL6A5. Additionally, BZXD exerts anti-inflammatory effects in the late stage mainly by increasing the expression of FASN and affecting fatty acid metabolism.

**Conclusion:**

BZXD exerts therapeutic effects on RA through multi-pathways in the early and late stages. This work may provide proteomic clues for treating RA by BZXD.

**Supplementary Information:**

The online version contains supplementary material available at 10.1186/s12906-022-03663-5.

## Introduction

Rheumatoid arthritis (RA) is a chronic, progressive, systemic autoimmune inflammatory disease [1]. Joint swelling and synovial inflammation are the dominant features in the early stage of RA [2], and continuous progression can lead to significant cartilage loss and bone erosion [3, 4]. If not treated timely and adequately, the disease can lead to joint deformities and loss of function [5]. Traditional disease-modifying antirheumatic drugs (DMARDs), biological agents or JAK inhibitors are critical in preventing RA but may have potentially adverse events [6]. Furthermore, these drugs are costly, and not all patients respond well to them [7]. Therefore, finding a safe, effective and cheap medicine has become an inevitable trend in RA research.

Chinese herbal medicine, the main part of traditional Chinese medicine (TCM), has been widely used in the therapy of RA and related disorders, called “Bi syndrome”, with promising outcomes from the ancient past of China [8]. In TCM theories, herbal formulations were administrated based on the identification of Chinese medicine patterns in the patient by analyzing the symptoms and characteristics, which emphasize individual treatment [9]. Herbal formulations have been reported to play important role in anti-inflammatory and anti-arthritic activity by influencing multiple pathways involved in the disease process [10, 11]. It is also considered valuable in treating RA because it can greatly improve the quality of life, prevent joint structural damage, and is highly tolerated by RA patients [12, 13]. Bi Zhong Xiao decoction (BZXD), a traditional Chinese herbal formula, has been used clinically for many years in our department to treat RA and has no significant side effects. In our previous studies, it was found that BZXD can improve the active movement of the joint and the ability of daily life in RA patients [14, 15]. It also decreases the degree of clinical symptoms of arthritis, paw volume, and arthritic score inhibits the inflammatory cytokines and the formation of synovial pannus, reducing the degradation of cartilage matrix in collagen-induced arthritis (CIA) rats [16, 17]. We also investigated the mechanisms by which the three important components of BZXD, paeoniflorin, ferulic acid, and coumaric acid inhibit inflammation and bone erosion in CIA rats [18–21]. However, the dynamic protein network related to the pathogenesis of the CIA rats at different developmental stages and the overall therapeutic mechanism of BZXD remains unknown.

Due to the complexity of systematic pathologies of RA, the exact pathogenesis mechanism of RA has not been fully understood. On the other hand, TCM is rather complex both in the effective chemical components and drug action mechanisms. Recent advances in proteomics provide a new opportunity to reveal the pathological mechanism of RA and the targeted proteins and networks related to drug actions. Especially, the development of the quantitative proteomic technique of isobaric tags for relative and absolute quantification (iTRAQ) with strong cation exchange (SCX)-reverse-phase liquid chromatography-tandem mass spectrometry (SCX-RPLC-MS/MS), which would be the more powerful proteomics methodologies in the pathogenesis mechanism and drug action of RA research [22–24].

To discover the targeted proteins and predict proteomic networks of BZXD anti-arthritic effect in CIA rats, iTRAQ-based quantitative proteomics was used in the present study to identify differentially expressed proteins (DEPs) in synovial tissue at different time points. Bioinformatics analysis was used to dynamically analyze the biological functions, pathways and interaction network of these DEPs. Furthermore, a major DEP was selectively validated by western blot.

## Material and methods

### Preparation of BZXD

BZXD consists of 9 herbs: Baihuasheshecao, Zhongjiefeng, Tengligen, Yiyiren, Danshen, Luoshiteng, Baishao, Fengxiantougucao and Gancao at a dry-weight ratio of 30:30:30:30:15:15:15:10:5 (Table 1). The herbs were purchased from Central South University Xiangya Hospital pharmacy and the quality was agreed with the People's Republic of China Pharmacopoeia (2015). They were authenticated by Prof. Suiyu Hu, Department of Chinese herbal medicine of Central South University (Changsha, China) and voucher specimens were deposited at the authors’ laboratory. Specimen numbers are presented in Table 1. We calculated the human daily herbal dose and converted it to a rat daily dose using the body surface area of an individual weighing 70 kg as previously described [17]; the daily dose per rat was thus 16.2 g/kg (crude drug/weight). These crude drugs were processed as follows: mixed, soaked for 1 h, and boiled twice in a multifunctional extraction tank (the amount of water added was 8 times and 10 times of crude drug, respectively, boiled for 1.5 h and 1 h, respectively). Combined the two filtrates to make a concentrated decoction, sealed and stored in a 4 °C refrigerator for later use.

**Table 1:** Compositions of Bi Zhong Xiao decoction

Plant name	Latin name	Chinese name	Medicinal part	Ratio	Specimen number
*Hedyotis diffusa Willd*	*Hedyotis Diffusae Herba*	Baihuasheshecao	Whole Herb	30	20,070,306
*Sarcandra glabra(Thunb.)Nakai*	*Herba Sarcandrae*	Zhongjiefeng	Whole Herb	30	19,062,901
*Actinidia arguta (Sieb. et Zucc) Planch. ex Miq*	*Actinidia Chinensis Planch*	Tengligen	Root	30	19,010,314
*Coix lacryma-jobi L.var.mayuen (Roman.) Stapf*	*Coicis Semen*	Yiyiren	Seed	30	20,072,307
*Salvia miltiorrhiza Bunge*	*Radix Salviae Miltiorrhizae*	Danshen	Root	15	20,080,410
*Trachelospermum jasminoides (Lindl.) Lem*	*Trachelospermumjasminoides*	Luoshiteng	Leaf and Stem	15	19,080,501
*Paeonia lactiflora Pall*	*Paeoniae Radix Alba*	Baishao	Root	15	20,041,711
*Speranskia tuberculate (Bunge) Baill*	*Impatiens Balsamina*	Fengxiantougucao	Whole Herb	10	19,091,208
*Glycyrrhiza uralensis Fisch*	*Radix Glycyrrhizae*	Gancao	Root	5	20,040,108

### Qualitative analysis of BZXD

According to the Pharmacopoeia of the People's Republic of China (2020 edition), six main components in BZXD have been identified. The quality stability and repeatability of these six marker components were detected by UPLC-MS/MS analysis method. We purchased the standard reference materials of Rosmarinic acid, Paeoniflorin, Glycyrrhizic acid, Salvianolic acid B, p-Coumaric acid, and Ferulic acid from Yuanye Bio-Technology Co., Ltd. (Shanghai, China). Qualitative analysis was carried out using an AB Sciex API 4000 LC/MS/MS system. Analytes were separated using a linear gradient program at a flow rate of 0.30 ml/min using a mobile phase consisting of 2 mmol/l ammonium acetate in water (A) and acetonitrile (B).

Precisely pipette 0.200 ml of thawed BZXD concentrate at 4 °C into a 2 ml brown PE tube, 0.6 ml mass spectrometry grade acetonitrile solution, and vortex for 5 min. Centrifuge at 13000r/min at 4 °C for 20 min. Take the supernatant and dilute it by 4 times, shake and mix, and filter through a 0.22-micron filter to be tested. The samples were detected and analyzed by negative ion electrospray tandem mass spectrometry (Supplementary Figure S1).

### Animals

Adult specific-pathogen-free Sprague Dawley rats of 6–7 weeks old, weighing 180 to 200 g, male and female each half, were provided by the Laboratory Animal Centre of Central South University (Changsha, China). All rats were housed in a well-ventilated room at 25 °C, with a 12 h dark/light cycle with free access to water and food for 1 week to adapt to the environment. All experimental protocols were approved by the Animal Ethics Committee of Central South University (No: 2020sydw0898) and were implemented in accordance with the Guide for the Care and Use of Laboratory Animals of the National Institutes of Health (NIH Publication No. 85–23, revised 1996).

### Collagen-induced arthritis (CIA) model

According to the Protocol For the Successful Induction of Collagen-Induced Arthritis (CIA) in Rats, Bovine type II collagen (2 mg/ml, Chondrex, Inc, Washington DC, USA) completely mixed with acetic acid and then mixed fully with complete Freund's adjuvant (CFA,1 mg/ml, Sigma-Aldrich Co., St Louis, MO, USA). On day 0, rats were injected intradermally at the base of the tail with 200 μg collagen/CFA emulsion for primary immunization. On day 7, rats were given 100 μg of collagen/incomplete Freund's adjuvant (IFA, 1 mg/ml, Sigma-Aldrich) emulsion for secondary immunization in the same way.

### Experimental groups and treatments

Rats were randomly assigned to 3 groups (*n* = 10 per group): Control, CIA and BZXD group. At 14 days after the primary immunization, rats in the BZXD group were administrated BZXD once a day for 28 days. The gavage volume for each rat was 4 mL, and the BZXD concentrate was diluted with distilled water to a dose of 16.2 g/kg/d based on daily body weight. Rats in the control and CIA groups were treated with 4 ml of saline solution in parallel.

### Arthritis assessments

**Fig. 1:**
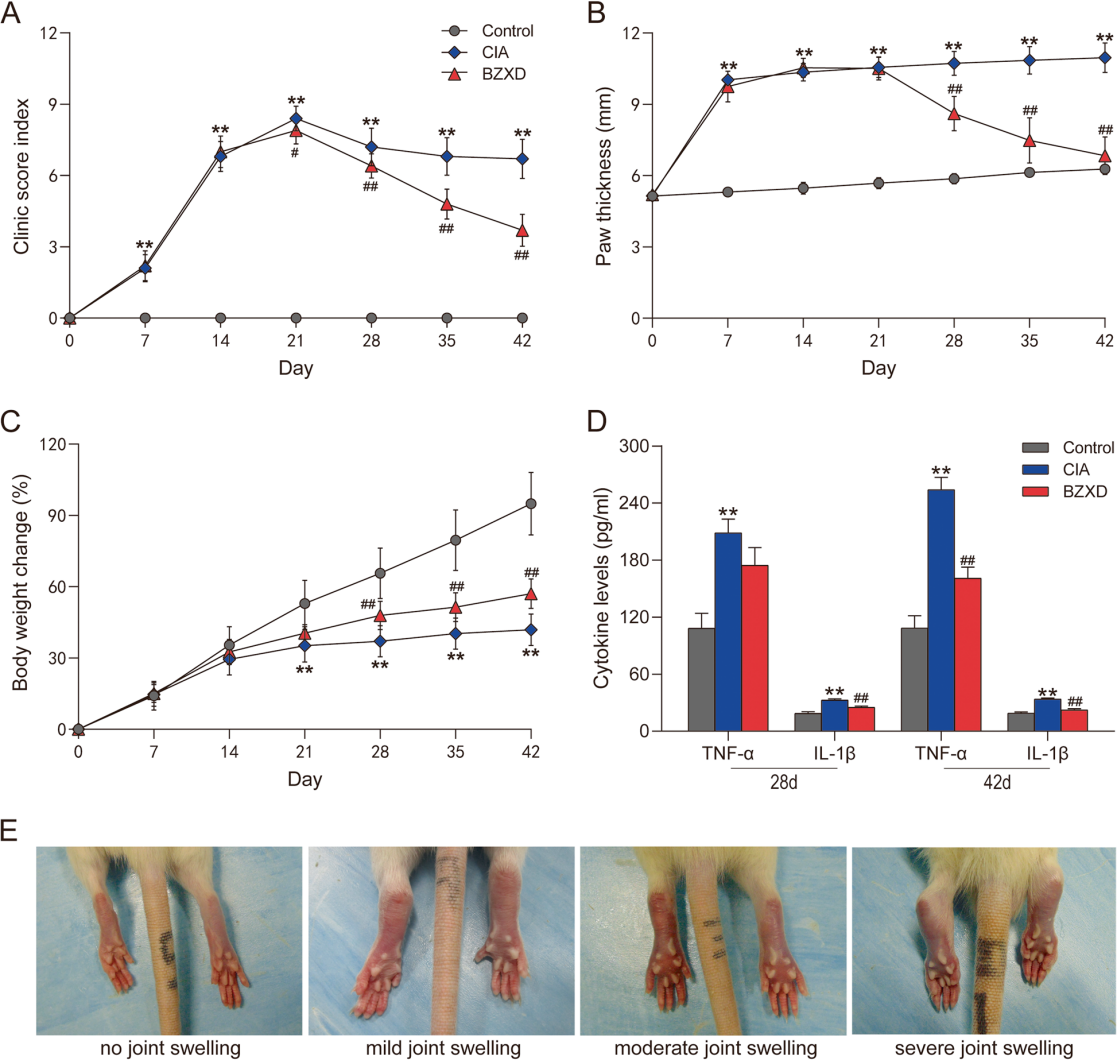
Effects of BZXD on the CIA rats. **A** Clinical scores index, **B** Paw thickness, **C** Body weight change was measured at 0, 7, 14, 21, 28, 35 and 42 days (*n* = 10 rats per group, two-way repeated-measures ANOVA followed by Fisher’s LSD test). **D** Serum inflammatory cytokines were measured 28 and 42 days by ELISA (*n* = 4 rats per group, one-way ANOVA followed by Fisher’s LSD test). **E** Representative images of different degrees of arthritis in rats. Data are presented as mean ± SD, *P < 0.05, **P < 0.01 CIA versus Control; #*P* < 0.05 ##*P* < 0.01 BZXD versus CIA

Clinical assessment, paw thickness, weight, serum inflammatory cytokine levels and histologic assessment were evaluated after arthritis onset. The incidence and severity of arthritis were evaluated using the clinical scoring system. The severity of arthritis in the foot was scored on a scale of 0–3 [25], where 0: no joint swelling; 1: mild joint swelling; 2: moderate joint swelling; 3: severe joint swelling (Fig. 1E). The disease score of the joints was calculated for each animal (maximum score of 12 per rat). At least one entire foot was swollen, including the toes, to be considered a successful arthritis induction. The thickness of the right paw of rats was measured with compasses and a millimeter ruler in the fixed position (1 cm anterior to the calcaneus) and body weight was monitored throughout the study.

### Sample collection and pretreatment

Rats were anesthetized by intraperitoneal injection of 3% pentobarbital sodium (60 mg/kg) and sacrificed by neck dislocation on day 28 and day 42. Collect blood samples for ELISA testing into vacuum tubes (red cap). All tubes were stored at room temperature for 2 h and centrifuged at 3000 r/min for 10 min at 4 °C to obtain serum, then stored at − 80 °C until analysis.

Cut the skin longitudinally along the midline of the knee joint until the knee joint is exposed. Lift the patella with tooth forceps and cut along the superior border of the patella to the femur. It is then separated down the sides of the patella to the tibia, and the knee joint cavity is opened. Finally, the free end was gently clamped with eye forceps, and the synovial tissue was completely cut off with a razor blade and stored at -80 °C.

Hind paws for hematoxylin–eosin (HE) staining were dissected and fixed in 10% neutral formalin for 24 h, decalcified in 14% ethylenediaminetetraacetic acid (EDTA) decalcification solution for 5 days, Neutralize in 5% sodium thiosulfate for 3 h, and embed in dehydrated paraffin.

### Enzyme-Linked Immunosorbent Assay (ELISA)

For detection of serum inflammatory cytokine, the serum obtained was added to the ELISA kit (specific for rats, Shanghai zcibio technology Co.,Ltd., China) to detect the levels of TNF-α and IL-1β. The optical densities were detected at 450 nm by a microplate reader (Rayto RT-6100, Rayto Life and Analytical Sciences Co., Ltd., China). The concentration of TNF-α and IL-1β were calculated with the standards curves.

### HE staining

For histologic assessment, longitudinal sections (5—6 μm) were cut from the center of the ankle joint and baked in 60 °C ovens for 30 min. Sections were then dewaxed in xylene, hydrated with graded ethanol, and stained with hematoxylin and eosin (Servicebio, China). Sections were observed by light microscope (CX21; Olympus, Tokyo, Japan) for pathological changes.

### Immunohistochemistry

Sections were deparaffinized with xylene, hydrated with graded ethanol, and antigen-retrieved with citrate antigen retrieval buffer (pH 6.0) in a microwave oven for 23 min. After natural cooling, the sections were placed in a 3% hydrogen peroxide solution (hydrogen peroxide: pure water = 1:9), and incubated at room temperature for 25 min in the dark to block endogenous peroxidase. Then, the paraffin sections were blocked for 2 h with 3% bovine serum albumin (BSA). The sections were incubated overnight at 4 °C with rabbit-anti-rat FASN antibody (10,624–1-AP, 1:500; Proteintech, USA) and rabbit-anti-rat COL3A1 antibody (GB111629, 1:500; Servicebio, China). They were subsequently incubated with HRP goat-anti-rabbit IgG (G3431-1, 1:200; Servicebio, China) for 50 min at room temperature and were exposed to diaminobenzidine (DAB) Peroxidase Substrate Kit reagent (Zsbio, Beijing, China) for 8 min. The sections were stained with hematoxylin (Servicebio, China) for 3 min. Finally, the sections were immersed in ethanol and xylene and fixed with neutral resin.

### Tissue preparation and protein extraction

The synovial tissues were dissolved in lysis buffer (8 M urea, 4% chaps, 30 mM HEPES, 1 mM phenylmethanesulfonyl fluoride, 2 mM EDTA and 10 mM DL-Dithiothreitol (DTT)) at 4 °C for 1 h, ultrasound (water-bath, 5 min) and then centrifuged at 20 000 rpm for 25 min. The supernatants were collected, added DTT, and incubated in a water bath (56 °C, 1 h); then, added quickly iodoacetamide to a final concentration of 55 mM and precipitated (-20 °C, 3 h) with the addition of precooled acetone, centrifuged (20 000 × g, 20 min, 4 °C). Finally, the proteins were solubilized in 0.5 M triethylammonium bicarbonate plus 0.1% Sodium dodecyl sulfate and measured their concentration by the Bradford protein assay kit (Ameresco). More details were detailedly performed in our previous study [20].

### iTRAQ method

Trypsin digestion and iTRAQ labeling were performed according to the manufacturer’s protocol (Applied Biosystems). Briefly, 100 μg protein of each pooled sample was reduced, alkylated and then digested overnight at 37 °C with trypsin. Apart (1 μl) of tryptic peptides was taken to detect digestion efficiency using Ultraflex TOF/TOF (Bruker, Germany). The tryptic peptide solution of each sample was labeled with iTRAQ reagents according to the iTRAQ Reagent Multiplex Kit protocol (Applied Biosystem). The tryptic peptide samples were mixed, fractionated and dried as described previously [20]. before further analysis. Identification and quantification of the iTRAQ-labeled samples were performed by Q Exactive LC–MS/MS (Thermo Scientific Co.). To reduce the influence of experimental variation on the proteomics analysis results, three independent MS/MS runs were performed on each sample [26].

The software used for data acquisition and quantitation was Proteome Discoverer software (Thermo Scientific version 1.3). The data sifted by Proteome Discoverer were used to identify proteins using Mascot (version 2.3.0, Matrix Science, London, UK) and the Uniprot database (UniProtKB Release 2020_06, 209,721,111 total entries) (http://www.uniprot.org/). The Mascot search parameters included trypsin, peptides digested with a maximum of one missed cleavage, fixed modification (carbamidomethylation of a cysteine residue), variable modifications (oxidation of methionine Gln-Pyro-Glu of N-term Q, and iTRAQ 8 plex modification of N terminal, K and Y), peptide tolerance 15 ppm, and the iTRAQ fragment tolerance (0.2 Da). Using these criteria 59,285 spectra were identified with 95% confidence. The differential expression proteins were identified with the following criteria: ≥ 2 peptides match, repeatedly identified in three replications, and an averaged ratio-fold change ≥ 1.2 or ≤ 0.84 between two groups.

### Bioinformatics analyses of DEPs

Venn diagram and dumbbell charts were exerted to exhibit the DEPs using OriginPro Origin® 2021 (version 9.8.0.200) and the ggpubr package in R, respectively. To explore the molecular mechanism, the DEPs were imported into DAVID Bioinformatics Resources v6.8 (https://david.ncifcrf.gov/) [27] to analyze Gene Ontology (GO, which contains a biological process (BP), cellular component (CC) and molecular function (MF)) and Kyoto Encyclopedia of Genes and Genomes (KEGG) pathways (www.kegg.jp/kegg/kegg1.html) [28]. The DEPs were also imported into STRING 11.0 (http://www.string-db.org/) to create protein–protein interaction (PPI) networks. Then, the PPI networks were visualized and analyzed using Cytoscape 3.7.2 [29]. Cytoscape is an open software that includes plug-ins for various visualization options and network analysis functions. To obtain the hub proteins in the PPI network, a topological analysis of the PPI network was performed by the Cytoscape plugin cytoHubba [30]. It’s a tool to evaluate the importance of nodes in biological networks by scoring and ranking nodes according to network characteristics. We calculated the maximum neighborhood component (MNC) scores of all nodes of the PPI network via the CytoHubba plugin, and a ranking table for each protein in the PPI network was obtained.

### Western blot

Western blot was performed in synovial tissues (Control d28, CIA d28, BZXD d28, Control d42, CIA d42 and BZXD d42, *n* = 4). Synovial tissues were lysed in RIPA buffer, followed by homogenizing mechanically. The homogenates were centrifuged at 12,000 rpm for 15 min at 4 °C. Protein samples were separated on 11% sodium dodecyl sulfate–polyacrylamide gel electrophoresis and then gel excision was cut according to the molecular weight of FASN (250-272KD) and β-actin (42KD). Cut a suitable nitrocellulose membrane according to the obtained gel size, transferred FASN at 300 mA for 150 min, and β-actin for 60 min. Following blocking with 5% skim milk, the transferred membranes were incubated overnight at 4 °C with rabbit-anti-rat FASN antibody (10,624–1-AP, 1:500; Proteintech, USA) and mouse-anti-rat β-actin antibody (66,009–1-Ig, 1:5000; Proteintech, USA), respectively. They were subsequently incubated with HRP goat-anti-rabbit IgG (SA00001-2, 1:6000; Proteintech, USA) and HRP goat-anti-mouse IgG (SA00001-1, 1:5000; Proteintech, USA) for 90 min at room temperature. Bands were visualized by electrochemiluminescence detection reagent (Thermo Scientific Pierce) and quantified by densitometry using an Image-Quant image analysis system (Storm Optical Scanner, Molecular Dynamics). The β-actin was detected simultaneously as a loading control.

### Statistical analysis

SPSS 26.0 software was used for statistical analysis. The data were expressed as the mean ± standard deviation (SD). Each time point index difference in the group was analyzed by the analysis of variance of repeated measurement design. Group comparison adopts an independent Student's t-test or one-way analysis of variance (ANOVA). A value of *P* < 0.05 was considered statistically significant.

## Results

### BZXD treatment ameliorates arthritis in CIA rats

Day 0 meant the day having the primary immunization and day 7 having booster immunization. On day 14, rats were administrated with BZXD or saline solution. As shown in Fig. 1, compared with the Control group, both CIA and BZXD groups showed a significant increase in the clinical score and paw swelling and a significant weight reduction. As shown in Fig. 1A and 1B, there was no difference in clinical score and paw swelling between groups before day 28. The onset of the significant difference in the clinical score and paw swelling was seen on day 28. The most significant weight change was observed on day 21 comparing the CIA versus BZXD group seen in Fig. 1C. These results implied that BZXD showed significant efficacy starting at 14 days of intervention. As was expected, significant increases in serum levels of IL-1β and TNF-α were observed both in the CIA and BZXD groups compared with the Control group (Fig. 1D). IL-1β serum levels were significantly reduced in the BZXD treated animals compared to the CIA rats on day 28 and day 42. However, we observed that the TNF-α serum level in the BZXD group was no different compared with the CIA group in the early stage (day 28), and was significantly reduced in the late stage (day 42). These results suggested that the mechanisms of the efficacy of BZXD in the early and late stages were different.

**Fig. 2:**
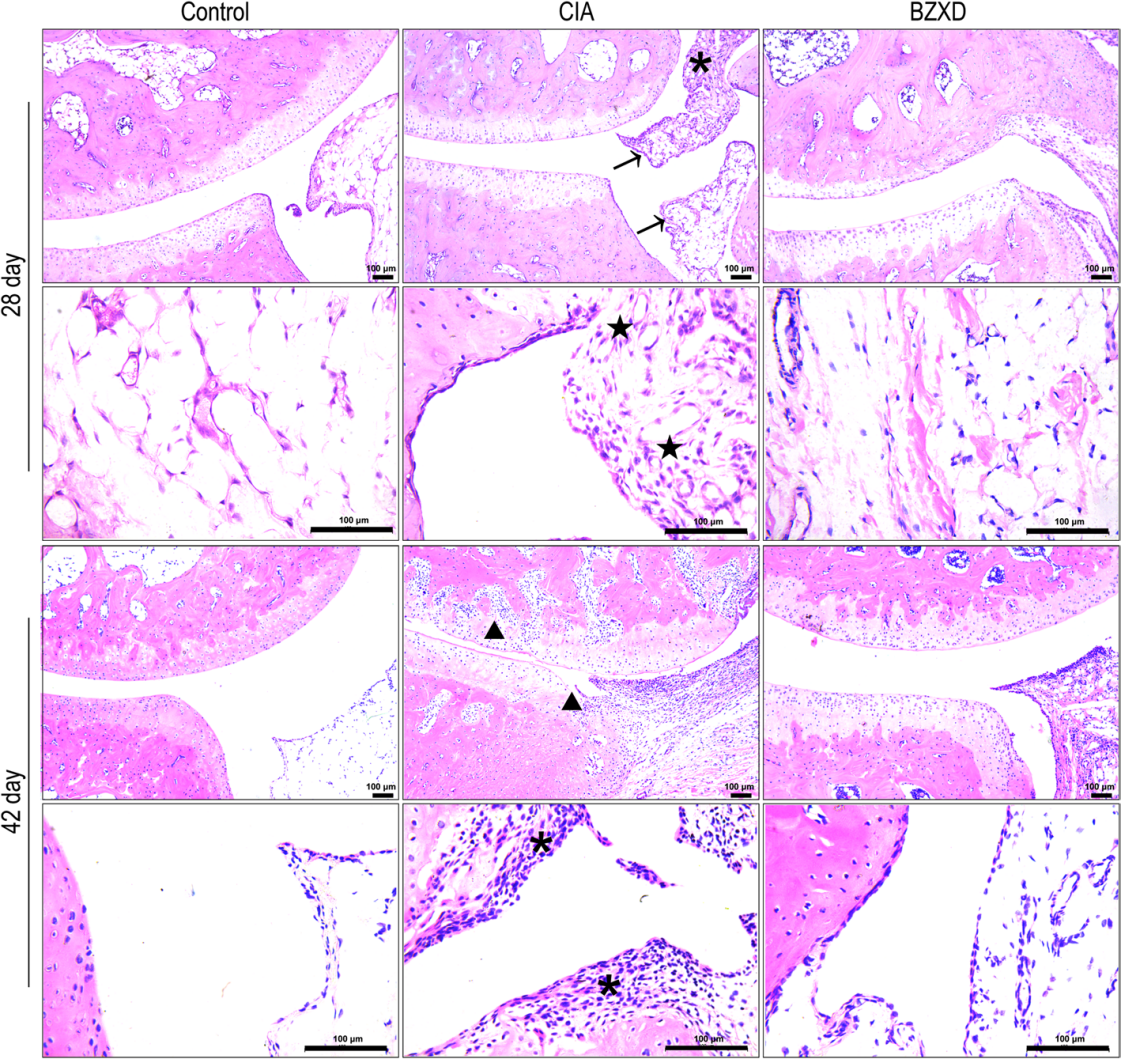
Histopathologic changes in the ankle joint of CIA rats affected by BZXD. Representative images of HE-stained (10X and 40X) in the ankle joint of rats; scale bar represents 100 μm (*n* = 5 rats per group). Asterisks indicate inflammatory cells infiltration; arrows show synovium hyperplasia; Pentagrams show synovial neoangiogenesis; Triangles show bone destruction

HE staining was used to observe the inflammatory cell infiltration in the synovial tissue of the hind ankle joints of rats. As shown in Fig. 2, the Control group had no signs of inflammation. In the early stage (day 28), some inflammatory cell infiltration and synovium hyperplasia were observed in the synovial tissues of rats in the CIA group; a large number of inflammatory cells and bone destruction could be seen in the late stage (day 42). On day 28 and day 42, the above symptoms in BZXD groups were alleviated compared with the CIA group. These results indicated that BZXD treatment ameliorated inflammation of the joints in CIA rats.

### iTRAQ analysis of DEPs

**Fig. 3:**
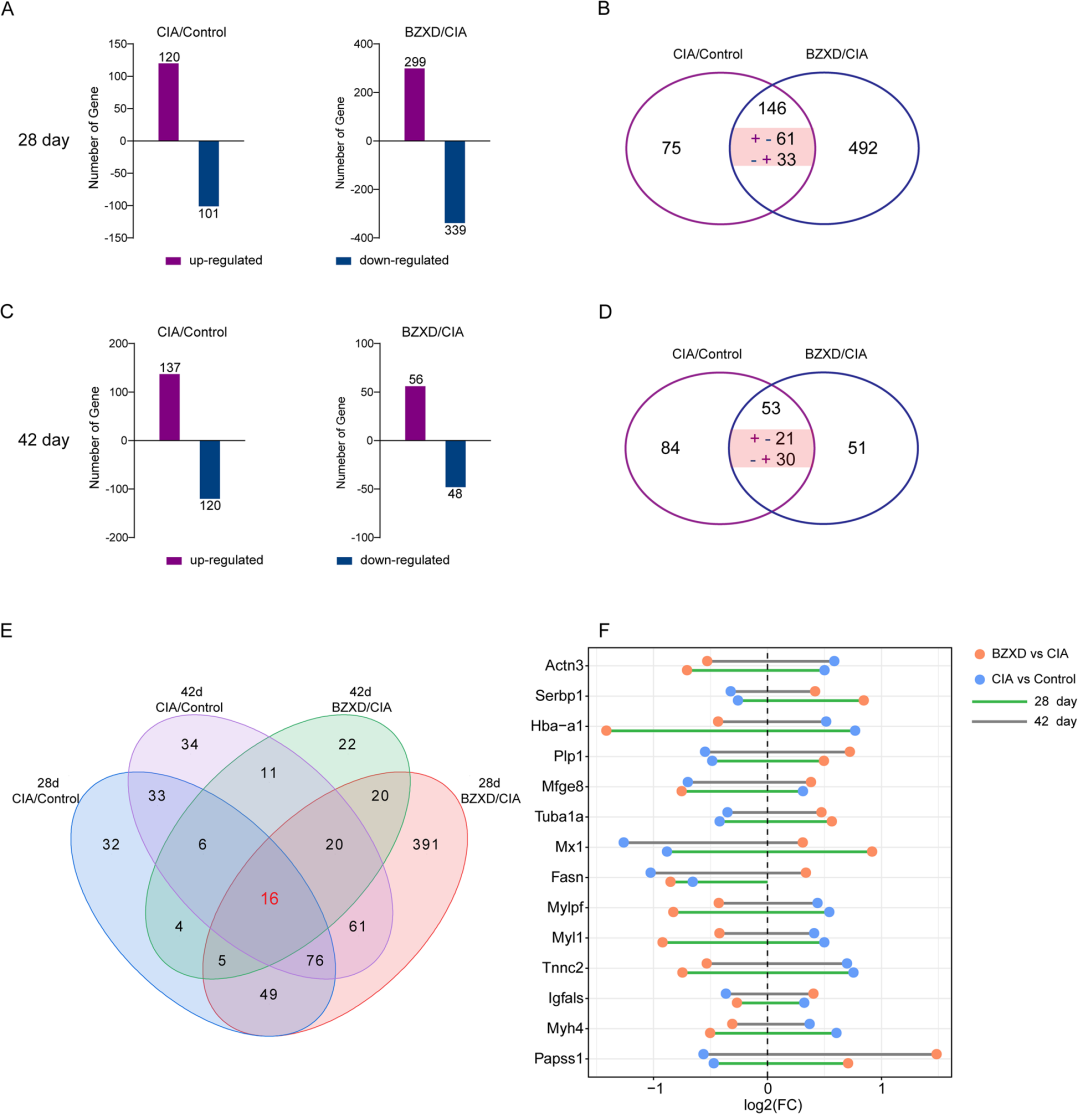
The differential proteins in CIA rats treated by BZXD. **A **and** C** The total number of identified DEPs from CIA/Control groups and BZXD/CIA groups on day 28 and day 42. **B **and** D** Venn diagram of DEPs and their overlap. Pink modules represent proteins that were reversely regulated by BZXD treatment in overlapped DEPs. The “ + ” indicates up-regulated proteins, while the “-” indicates down-regulated proteins. The “ + -” indicates proteins were up-regulated in the CIA/Control group but down-regulated in the BZXD/CIA group. The “- + ” indicates proteins were down-regulated in CIA/Control groups but up-regulated in BZXD/CIA groups. **E** Venn diagrams of the potential proteins associated with CIA and BZXD treatment on day 28 and day 42. **F** The fold change dumbbell charts of potential proteins

To define protein alterations changed in disease progression, we performed LC–MS/MS of synovial tissues dissected from all groups. DEPs were screened by the criteria (≥ 1.2 or ≤ 0.84) as described above. On day 28, In the CIA/Control group, 221 DEPs were identified (120 up-regulated and 101 down-regulated); in BZXD/CIA group, 638 DEPs were appraised (299 up-regulated and 399 down-regulated) (Fig. 3A). On day 42, 257 DEPs (137 up-regulated and 120 down-regulated) were identified in the CIA/Control group, and 104 DEPs (56 up-regulated and 48 down-regulated) in the BZXD/CIA group (Fig. 3C). In this study, the proteins that can be regulated by BZXD must meet the following two conditions: 1. The proteins are differentially expressed in the CIA/Control group and BZXD/CIA group (overlapping DEPs) 2. The expression trend of proteins in the CIA/Control group and BZXD/CIA group were opposite (+—proteins or—+ proteins). 146 overlapping DEPs were identified in both the CIA/Control group and BZXD/CIA group on day 28, and 53 DEPs on day 42 (Fig. 3B and D, Tables S1 and S2). Among these DEPs, 94 proteins were regulated by BZXD treatment on day 28, and 51 on day 42 (pink modules in Fig. 3B and D). The fold changes of 54 DEPs were consistent in the CIA/Control group and BZXD/CIA group on day 28, and 2 on day 42. This indicated that BZXD failed to regulate these proteins.

In addition, 16 overlapping proteins were differentially expressed between the CIA/Control group and BZXD/CIA group at two-time points (day 28 and 42) (Fig. 3E). To visualize their fold change in each individual, we plotted dumbbell charts. Figure 3F shows that 14 candidate proteins were reversely changed in the CIA/Control group and BZXD/CIA group. The fold changes of GW7_03778 and GW7_15073 were consistent in CIA/Control and BZXD/CIA groups regardless of day 28 or day 42, so they were removed.

### Bioinformatics analysis of DEPs

#### Functional classification of DEPs

**Fig. 4:**
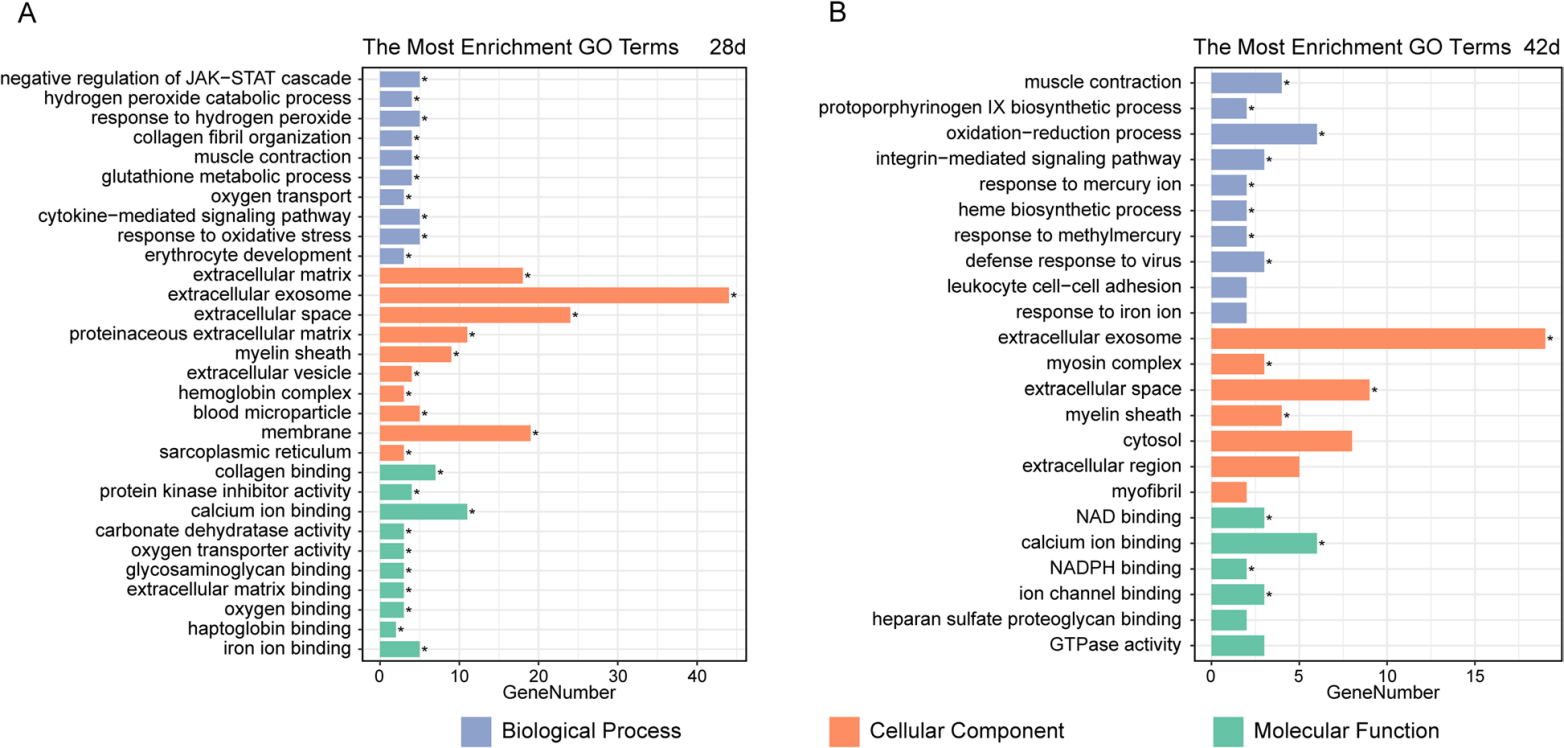
The functional classification of differentially expressed and reversely regulated by BZXD treatment proteins. **A **and** B** GO analysis of the 94 DEPs and 51 DEPs after being imported to DAVID on day 28 and day 42, respectively. *p < 0.05

To acquire functional information induced by BZXD, we imported BZXD-regulated proteins in DAVID with 94 on day 28 and 51 on day 42. GO annotations of the differential proteins (41 biological processes, 15 cellular components and 20 molecular functions) were obtained on day 28. On day 42, we obtained 13 biological processes, 7 cellular components and 6 molecular functions of DEPs. Subsequently, we used the R ggplot2 package to visualize the top 10 GO enrichment terms (Fig. 4A and B).

In the biological process, the proteins were located in negative regulation of the JAK-STAT cascade, collagen fibril organization, muscle contraction, cytokine-mediated signaling pathway, and integrin-mediated signaling pathway. For cellular components, DEPs focused on extracellular matrix, extracellular exosome, extracellular space, and proteinaceous extracellular matrix. As for molecular function, DEPs were associated with collagen-binding, protein kinase inhibitor activity, NAD binding, and calcium ion binding.

#### KEGG pathway analysis of DEPs

**Fig. 5:**
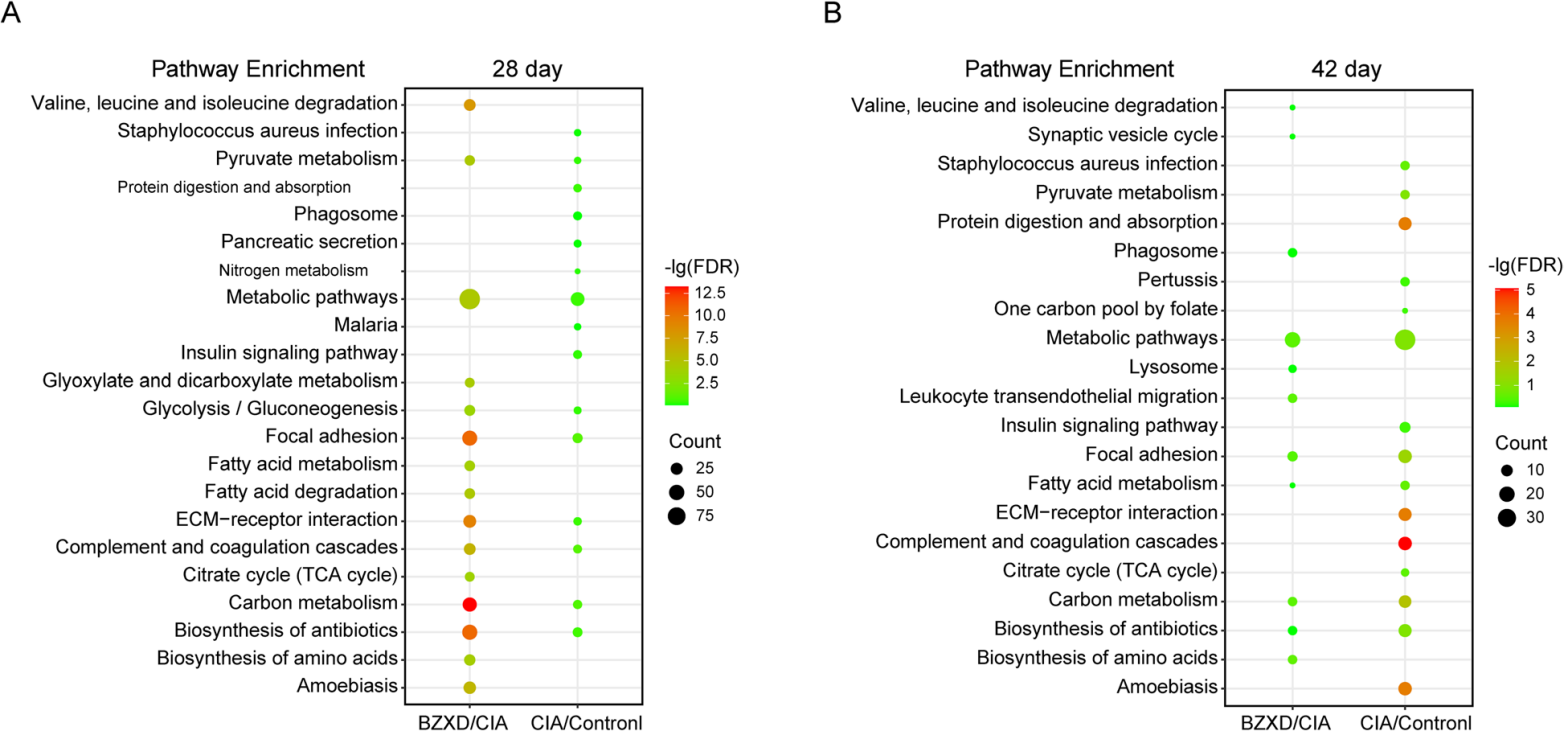
The pathways of DEPs of CIA/Control groups and BZXD/CIA groups on day 28 and day 42. **A **and** B** The KEGG pathways enrichment analysis by DAVID. The color depth shows the -lg (FDR) values. Bubble size depicts the number of proteins identified in the pathway

To uncover the functional association between the CIA/Control group and BZXD/CIA group differential proteins on day 28 and day 42, respectively. We conducted a KEGG analysis using DAVID. On day 28, 20 pathways were enriched at protein expression in the CIA vs Control, and 58 pathways in the BZXD vs CIA comparisons. 24 pathways were enriched in the CIA vs Control and 11 pathways in the BZXD vs CIA comparisons on day 42. The top 15 significant KEGG pathways were listed in Fig. 5, there were considerable overlaps of pathways between the two groups, suggesting BZXD may ameliorate joint inflammation by regulating these pathways (Table 2). Overall, the top enriched biological pathways for both day 28 and day 42 included focal adhesion, carbon metabolism, biosynthesis of antibiotics and metabolic pathways. In addition, the complement and coagulation cascades, ECM-receptor interaction, glycolysis/gluconeogenesis and pyruvate metabolism were enriched significantly in the early stage (day 28). The KEGG pathway analysis also revealed that the identified DEPs were mainly involved in fatty acid metabolism in the late stage (day 42).

**Table 2:** Overlapping KEGG pathways between the two groups

**KEGG Pathways**	**Common Genes**	**CIA/Control**		**BZXD/CIA**	
		***P***Value	**Count**	***P***Value	**Count**
**28 Day**					
Focal adhesion	MYLPF, ACTN3, CAV1, COL5A2, COL6A1, CHAD, TLN2	0.0011	11	0.0000	39
Carbon metabolism	HK3, PC, ME1, PGP, ENO2	0.0021	8	0.0000	33
Biosynthesis of antibiotics	ENO2	0.0051	10	0.0000	40
ECM-receptor interaction	COL5A2, COL6A1, CHAD	0.0102	6	0.0000	24
Glycolysis / Gluconeogenesis	HK3, ALDH2, ENO2	0.0199	5	0.0000	14
Pyruvate metabolism	PC, ALDH2, ME1	0.0211	4	0.0000	12
Complement and coagulation cascades	CFD, KLKB1, KNG2, KNG1, C2	0.0007	7	0.0000	18
Metabolic pathways	HIBADH, GDA, HEXB, PYGM, NT5C2, ENO2, PAPSS1, HK3, ALDH2, ME1, GGT5, CKM, ACLY, PC, FASN, PGP, PLPP3	0.0113	30	0.0000	96
**42 Day**					
Focal adhesion	MYLPF, COL3A1, ACTN3, COL6A5	0.0012	12	0.0139	6
Carbon metabolism	RPIA, PHGDH, GPT	0.0003	10	0.0092	5
Biosynthesis of antibiotics	RPIA, PHGDH	0.0053	11	0.0620	5
Fatty acid metabolism	FASN, ACADSB	0.0149	5	0.0524	3
Metabolic pathways	AHCYL1, GPT, AKR1B8, PAPSS1, ALAD, UROD, PHGDH, FDPS, ACADSB, RPIA, FASN	0.0046	36	0.0098	17

#### PPI analysis of DEPs

**Fig. 6:**
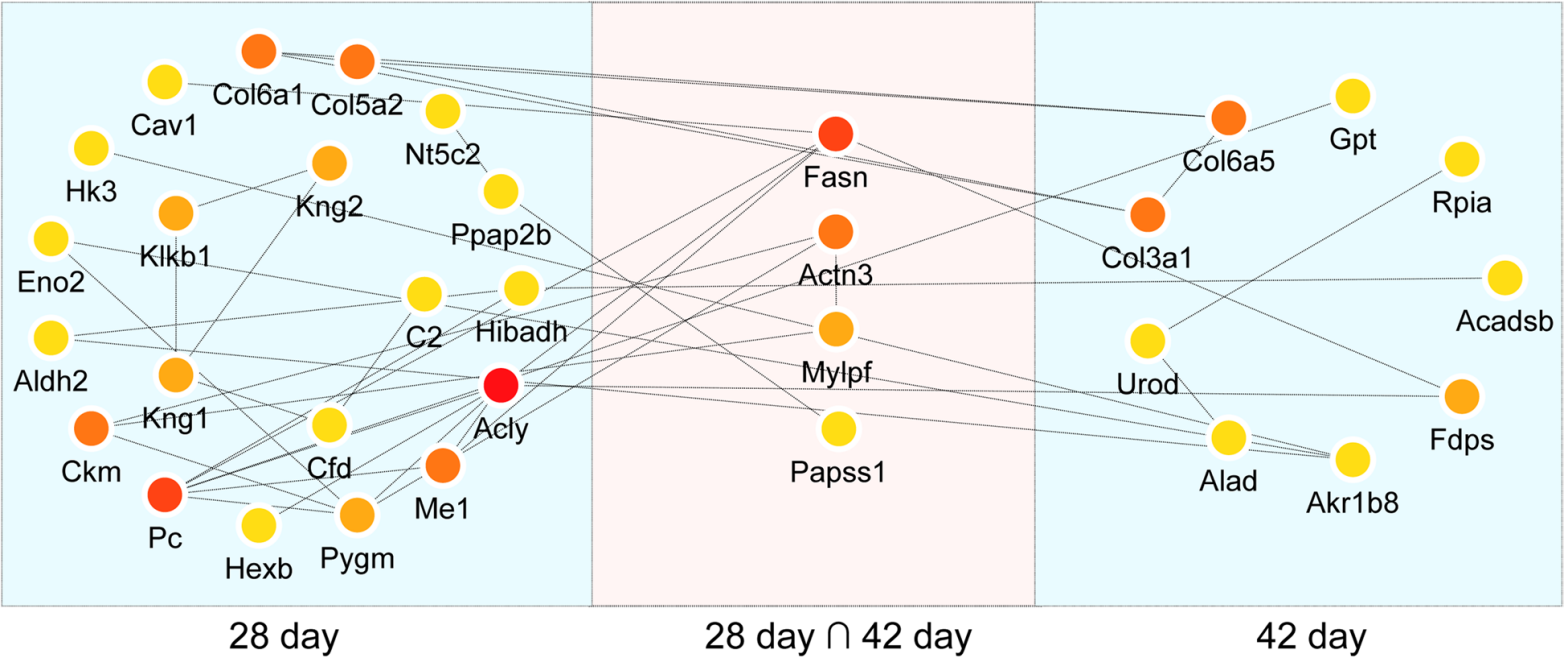
The PPI network of differentially expressed proteins. The PPI analysis of the 41 DEPs enriched in important pathways by STRING. Color depth represents the level of significance, with deeper colors indicating greater significance. The cyan modules showed that proteins were reversely regulated by BZXD on day 28 or day 42. The light pink module showed that proteins were reversely regulated by BZXD both on days 28 and 42

We used the STRING database to determine the relationship between 41 DEPs, which were enriched in the above pathways. DEPs with a combined protein interaction score > 0.4 were selected and used to construct a PPI network. We visualized the PPI network by Cytoscape. Then, to identify the hub proteins of BZXD against CIA, we performed the topological analysis of the PPI network via the Cytoscape plugin cytoHubba. It’s a tool to evaluate the importance of nodes in biological networks by scoring and ranking nodes according to network characteristics. We calculated the maximum neighborhood component (MNC) scores of all nodes of the PPI network via the CytoHubba plugin. The top three proteins were ACLY, FASN, PC (Table S3). Figure 6 shows the overall relationships within DEPs. Color depth represents the level of significance, with deeper colors indicating greater significance.

#### Validation of DEPs

**Fig. 7:**
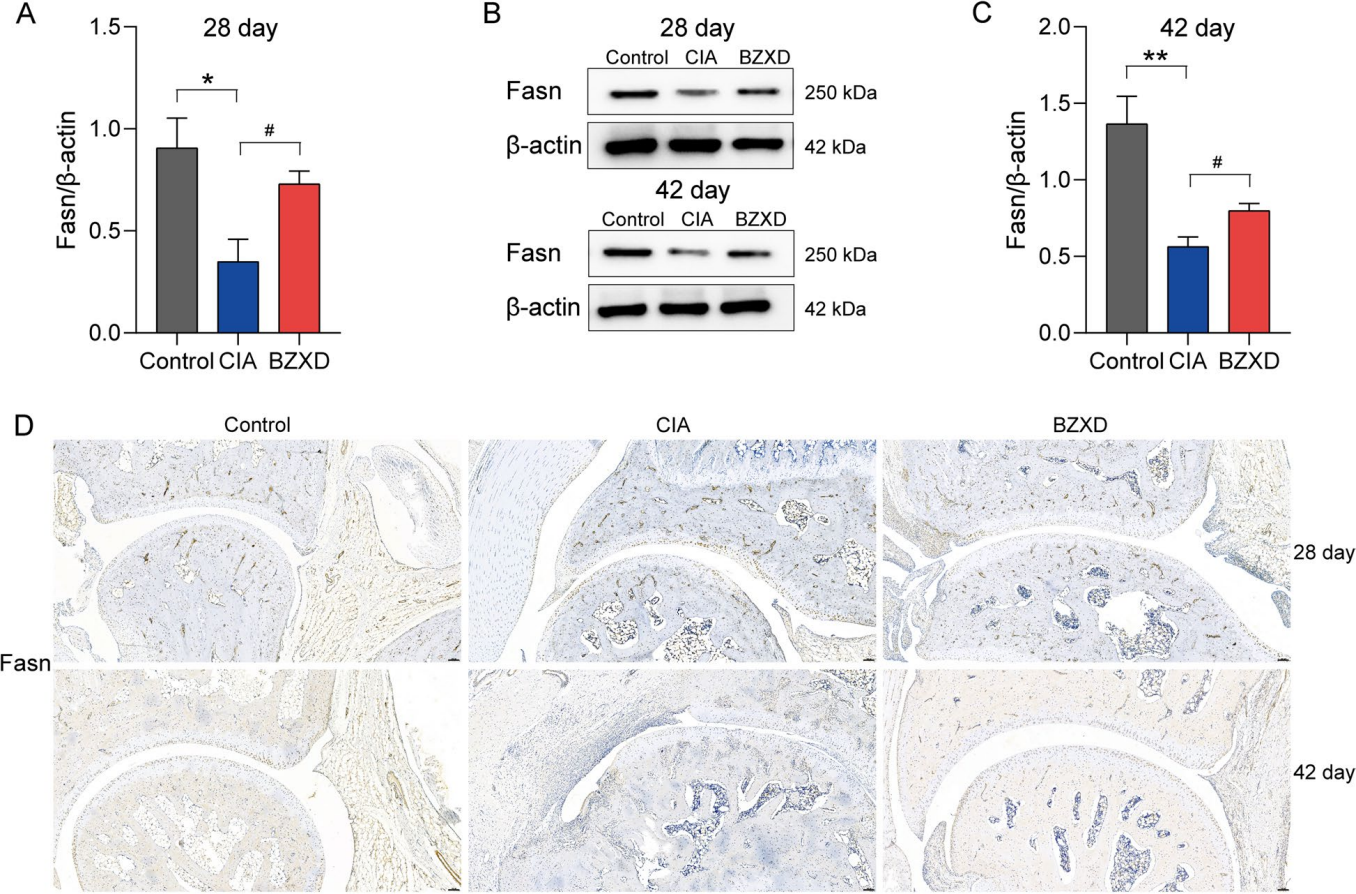
Effects of BZXD on the expression levels of FASN in CIA rats. Representative western blots of FASN (**A **and** C**) and their quantifications (**B**) among Control, CIA and BZXD groups on day 28 and day 42. Representative images of FASN immunoreactivity (10X) in the ankle joint of rats; scale bar represents 100 μm (*n* = 3 rats per group) (**D**). Data are presented as mean ± SD, *n* = 4 rats per group, student's t-test. *P < 0.05, **P < 0.01 CIA versus Control; #*P* < 0.05 ##*P* < 0.01 BZXD versus CIA

FASN ranked second in importance for MNC topological analysis, suggesting that it may be a hub functional protein. In addition, Fig. 6 showed that FASN, ACTN3, MYPLF, and PAPSS1 were proteins that were reversely regulated by BZXD on day 28 and day 42, and the color of FASN is the most depth among these proteins. Therefore, we chose FASN to verify the reliability of quantitative proteomics by western blot and immunohistochemistry. On day 28 and day 42, western blot analyses showed that FASN was notedly down-regulated, and BZXD reversed its expressions significantly (Fig. 7A-C). The FASN immunohistochemical analysis of rat ankle joints were shown in Fig. 7D. The FASN immunoreactivity in the control group was significantly aggregated in the synovial tissue, while the synovial tissue of the CIA group was more infiltrated by inflammatory cells. Overall, The positive FASN immunoreactivity (brown staining) was significantly decreased in the CIA group compared with the Control group; the brown staining in the BZXD group was darker than that in the CIA group, indicating a regulatory effect of BZXD on FASN. Proteomic data also showed that FASN was down-regulated in the CIA/Control group, and up-regulated in the BZXD/CIA group. The result of the western blot and immunohistochemistry were consistent with proteomic data.

## Discussion

In this study, we demonstrated that BZXD could effectively inhibit joint swelling, synovial inflammation, and joint destruction in CIA rats. BZXD significantly reduced the level of inflammatory factor IL-1β in the early and late stages. Although the inflammatory factor TNF-α decreased in the early stage, the difference was not statistically significant. In the late stage, BZXD significantly decreased TNF-α levels with statistical significance. This suggests that in addition to disease progression over time, the regulatory mechanisms of BZXD in the early and late stages will be distinct and dynamic. Given the many complex components of BZXD, we intend to carry out proteomic studies on synovial tissue in the early and late stages of CIA rats. Bioinformatic analysis identified several signaling pathways including the metabolic pathways, complement and coagulation cascades, focal adhesion, ECM-receptor interaction, glycolysis/gluconeogenesis, pyruvate metabolism and fatty acid metabolism. We analyzed the DEPs within the above-mentioned signaling pathways and verified a protein with a high node degree and important functions by western blot. Overall, BZXD has played a systemic role, enriching our understanding of the mechanism of treating RA.

ECM-receptor interaction is an important signaling pathway in the progression of RA, which is significantly enriched in the early stage. Extracellular matrices are composed of collagen, elastin, fibronectin, laminins, proteoglycans, hyaluronan, and several glycoproteins such as matricellular proteins. Collagen is the oldest and most abundant component in extracellular matrices, which is mainly involved in the formation of fibrillar and microfibrillar networks of the extracellular matrices and other structures of the extracellular matrices [31]. In RA, collagen network disruption, and overexpression of the proinflammatory cytokine TNF-α can lead to an imbalance between anabolic and catabolic processes of extracellular matrices, ultimately leading to cartilage destruction [32, 33]. Previous studies have shown that COL3A1 is increased in osteoarthritic cartilage compared to normal cartilage, and its expression may correlate with the radiographic severity of osteoarthritis [34]. Our immunohistochemical results showed that COL3A1 was increased in CIA cartilage compared with normal cartilage, more pronounced in the late stage (Supplementary Figure S2). This indicated that COL3A1 expression increased over time and correlated with arthritis severity in CIA rats. CIA rats also had severe bone destruction in the late stage as observed in HE. In addition, studies have confirmed that IL-1β could induce the upregulated secretion of COL3A1 from the human synoviocyte [35]. Our research demonstrated that BZXD treatment inhibited the expression of IL-1β and decreased COL3A1 expression. Moreover, COL5A2 and COL6A1 were also demonstrated as upregulated genes associated with a pathological process of subchondral bone in osteoarthritis [36, 37]. In our study, PPI analysis showed that multiple collagen subtypes (COL3A1, COL5A2, COL6A1, and COL6A5) are connected closely with high degrees. These collagens were both upregulated in the CIA group, which indicates that these collagens have implications for the progression of RA. BZXD can downregulate the expression of these collagens, which may be the molecular mechanism of BZXD treating RA. The production of TNF-α also leads to the release of collagen, and matrix metalloproteinases [38], but BZXD can reduce the expression of TNF-α in our study. Therefore, BZXD may have potentially beneficial effects on preventing cartilage degradation by regulating collagen protein and TNF-α in CIA rats.

The focal adhesion pathway is a complex and multi-intersection network, which is closely related to the ECM receptor interaction pathway. It can regulate cell movement, proliferation, differentiation, expression and apoptosis [39, 40]. The formation of specific adhesion points focal adhesion kinase at the cell membrane-cytoplasm contact is an important step in the cell–matrix interaction [39]. Some studies have shown that the focal adhesion kinase family kinases are overexpressed in RA synovial tissues, which promote synovial fibroblast invasion and migration [41, 42]. Thence, the focal adhesion pathway plays an important role in the occurrence, development and progression of RA. This study also suggested that the focal adhesion pathway was significantly enriched in both early and late stages. DEPs (MYLPF, ACTN3, CAV1, CHAD, TLN2, COL3A1, COL5A2, COL6A1 and COL6A5) participated in the focal adhesion pathway. CAV1 is the main component of the caveolae structure, which promotes endocytosis, cell signaling, and endothelial-mediated inflammation [43]. Research has shown that silencing of CAV1 significantly decreased cell proliferation and promoted apoptosis in RA fibroblast-like synoviocytes [44]. Our research shows that BZXD reduces the expression of CAV1 in CIA rats, indicating that CAV1 may be a potential target for BZXD to exert therapeutic effects. In addition, CHAD is mainly localized in the territorial matrix of the deeper parts of the articular cartilage [45]. It mediates intracellular signal transduction between chondrocytes and the ECM through binding to the α2β1 integrin [46, 47]. CHAD also binds collagen type II and type VI and can inhibit the spreading of chondrocytes [48, 49]. CHAD plays a critical role in regulating linkages between collagens and other ECM molecules in vivo, as well as the communication between chondrocytes and their surrounding matrices [49]. In our study, the BZXD group reduced the expression of CHAD compared with the CIA group. Therefore, we speculated that adhesion-related proteins may be an important feature of BZXD in treating RA.

The microenvironment of RA synovial tissue accumulates a variety of cytokines, adipocytokines and metabolic intermediates, which make a variety of metabolic pathways dysregulated, including glycolysis, tricarboxylic acid cycle, pentose phosphate pathway, and lipid metabolism. The dysregulation of metabolic pathways deteriorates pro-inflammatory immune responses and chronic inflammation [50]. Some studies have shown that glycolysis is increased in the RA synovial tissues and leads to persistent synovial inflammation and joint damage (31,921,178, 32,938,830, 26,815,411). In addition, the end product of glycolysis is pyruvate, which could stimulate the expression of vascular endothelial growth factor (VEGF) mRNA [51]. In our previous study [52], we found that the expression of VEGF was increased in the synovial tissues of CIA rats, while BZXD significantly decreased its level, indicating that BZXD maybe reduces the symptoms of RA by modulating the pyruvate metabolism. This needs further study. In this study, metabolic pathways, pyruvate metabolism, and glycolysis/gluconeogenesis were disturbed in the CIA group, while BZXD regulated these disordered metabolic pathways back to normal.

Fatty acid metabolism is a dynamic process of anabolic and catabolic reactions that maintain energy homeostasis [53]. The regulation of fatty acid synthesis is closely related to the physiological and pathophysiological processes regulated by immune cells [54]. Studies have also determined the role of fatty acids in immune response and inflammation, that is saturated fatty acids promote inflammation, while polyunsaturated fatty acids play an anti-inflammatory effect [55]. In our study, KEGG showed that the CIA rat group was significantly enriched in fatty acid metabolism, and BZXD may exert anti-inflammatory effects by regulating fatty acid metabolism. Furthermore, FASN acts as a central regulator of lipid metabolism and is overexpressed in inflammation and the immune system [56–58]. Interestingly, the previous research [59] reported that FASN is specifically low-expressed in the synovial tissue of CIA rats. This is consistent with our findings. Proteomic data showed that FASN was downregulated in the CIA/Control group, which was also confirmed by western blot and immunohistochemistry. Following secretion of the pro-inflammatory cytokines TNF-α, adipocytes reduce the secretion of adiponectin, resulting in a decrease in lipid accumulation, thereby inhibiting the expression of FASN in arthritic rats [59]. This result was confirmed by our FSAN immunohistochemistry. FASN immunohistochemistry showed obvious inflammatory cell infiltration in the synovial tissue of the CIA group, and the positive immunoreactivity of FASN in the CIA group was significantly lower than that in the control group (Fig. 7D). In addition, proteomic data showed that FASN was a down-regulated differentially expressed protein in the BZXD/CIA group, which was consistent with the CIA/control group (Fig. 3F). This seems to suggest that BZXD can regulate the expression of FASN, but fails to reverse its expression. However, Western blotting and immunohistochemistry observed that BZXD down-regulated the expression of FASN. In the late stage, proteomic data, western blot, and immunohistochemical results all showed that BZXD significantly down-regulated the expression of FASN. This suggests that the therapeutic mechanism of BZXD on CIA rats may be different in the early and late stages. Coincidentally, this result was consistent with BZXD reducing the expression of the proinflammatory cytokine TNF-α, which was not significant in the early stage and significant in the late stage. We speculate that the therapeutic effect of BZXD treatment on regulating the expression of FASN is also related to the secretion of TNF-α, but this needs to be confirmed by further experiments.

Our current study has some limitations. This work only provides proteomic clues about the different mechanisms by which BZXD treats the early and late stages of RA. However, it provides potential targets and signaling pathways for follow-up studies. Therefore, our future work will focus on the specific mechanisms of signaling and gene changes after BZXD treatment.

## Conclusions

In summary, this proteomics work focuses on an overview of the multiple functions regulated by BZXD for RA treatment. These observations give us a broad understanding of the early and late treatment effects of BZXD on RA.

## Supplementary Information

**Additional file 1:** **Supplementary Figure S1.** UPLC-MS/MS analysis of BZXD components. UPLC-MS/MS TIC chromatograms of six major components (1: Paeoniflorin, 2: Rosmarinic acid, 3: Salvianolic acid B, 4: Glycyrrhizic acid, 5: Ferulic acid, 6: p-Coumaric acid) were detected in BZXD in the negative ESI mode. **Supplementary Figure S2.** Representative images of immunoreactivities (20X) in the ankle joint of rats; scale bar represents 50 μm. **Supplementary Figure S3.** (A-D) Original image of all blots with visible edges in Fig. 7. The figures a-d were cutting blots. The red box represented the original blot area used in Fig. 7. 

**Additional file 2:** **Table S1. **146 overlapping DEPs in the CIA/Control group and BZXD/CIA group.

**Additional file 3:** **Table S2. **53 overlapping DEPs in the CIA/Control group and BZXD/CIA group.

**Additional file 4:** **Table S3. **Top 33 in PPI network ranked by MNC method.

## Data Availability

Data of this study are included in the article and the primary data can be provided by the corresponding author.
