# Supplementary material for: Quantitative proteomic analysis of Bi Zhong Xiao decoction against collagen-induced arthritis rats in the early and late stages
Source: BMC Complement Med Ther. 2022 Jul 13;22:186. doi: 10.1186/s12906-022-03663-5 (PMC9281147; doi:10.1186/s12906-022-03663-5)
Supplement: Supplementary file 1 — Additional file 1: Supplementary Figure S1. UPLC-MS/MS analysis of BZXD components. UPLC-MS/MS TIC chromatograms of six major components (1: Paeoniflorin, 2: Rosmarinic acid, 3: Salvianolic acid B, 4: Glycyrrhizic acid, 5: Ferulic acid, 6: p-Coumaric acid) were detected in BZXD in the negative ESI mode. Supplementary Figure S2. Representative images of immunoreactivities (20X) in the ankle joint of rats; scale bar represents 50 μm. Supplementary Figure S3. (A-D) Original image of all blots with visible edges in Fig. 7. The figures a-d were cutting blots. The red box represented the original blot area used in Fig. 7. [file 12906_2022_3663_MOESM1_ESM.pdf]

Supplementary Figures

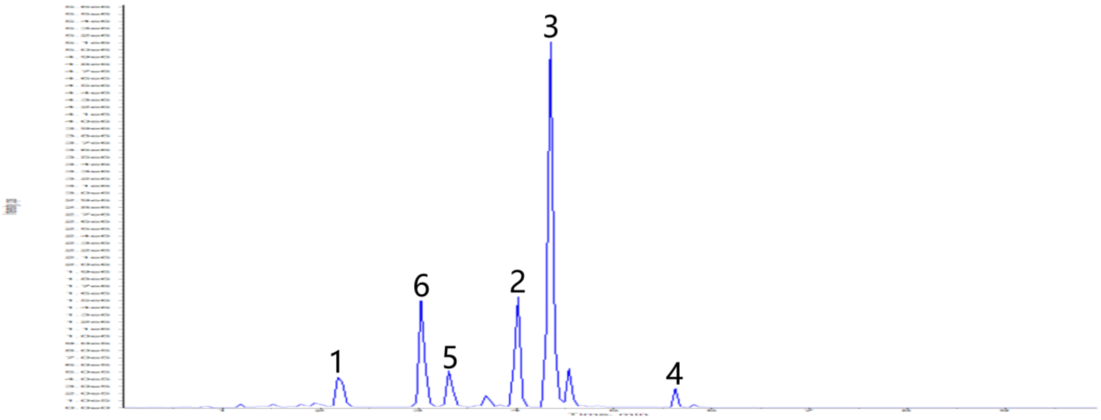

Supplementary Figure S1 | UPLC-MS/MS analysis of BZXD components. UPLC-MS/MS TIC chromatograms of six major components (1: Paeoniflorin, 2: Rosmarinic acid, 3: Salvianolic acid B, 4: Glycyrrhizic acid, 5: Ferulic acid, 6: p-Coumaric acid) were detected in BZXD in the negative ESI mode.

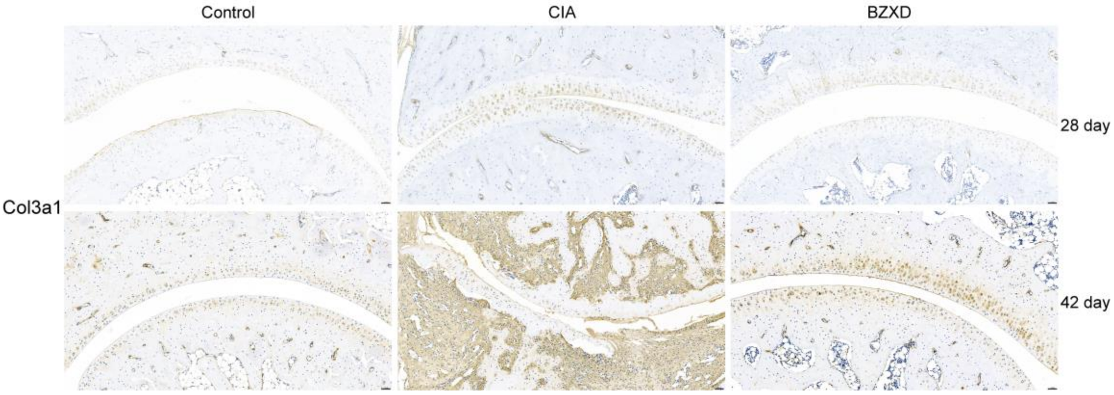

Supplementary Figure S2 | Representative images of immunoreactivities (20X) in the ankle joint of rats; scale bar represents 50 μm.

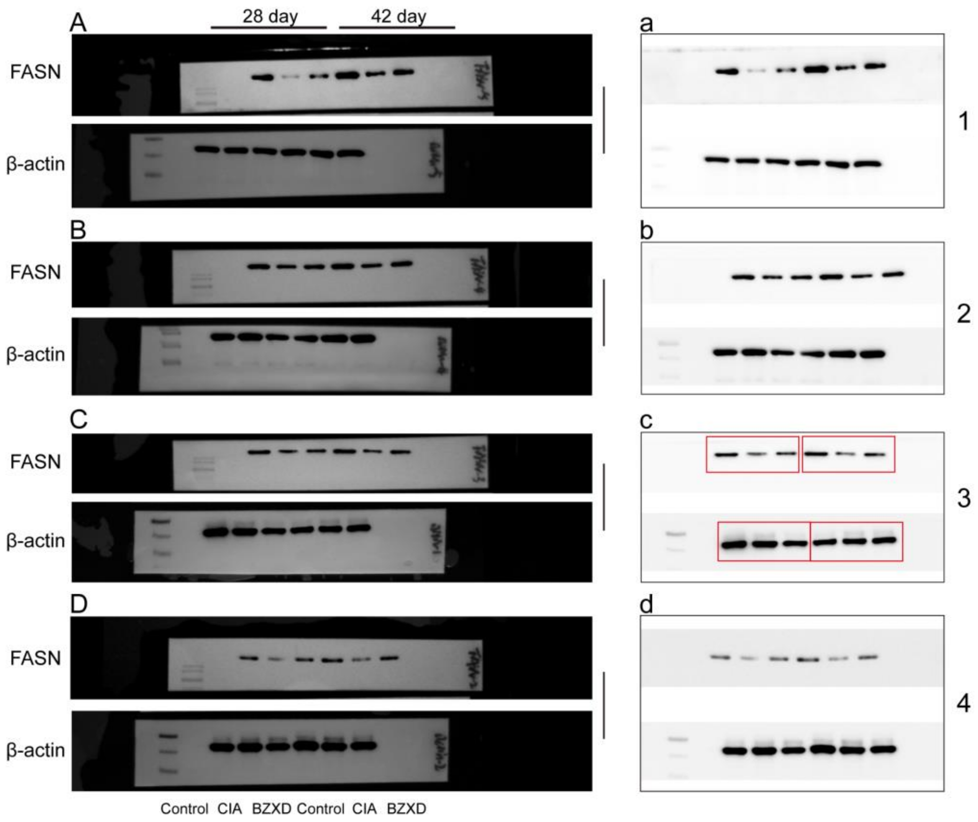

Supplementary Figure S3 | (A-D) Original image of all blots with visible edges in **Fig. 7**. The figures **a-d** were cutting blots. The red box represented the original blot area used in **Figure 7**.
