# Supplementary material for: Quantitative proteomic analysis of Bi Zhong Xiao decoction against collagen-induced arthritis rats in the early and late stages
Source: BMC Complement Med Ther. 2022 Jul 13;22:186. doi: 10.1186/s12906-022-03663-5 (PMC9281147; doi:10.1186/s12906-022-03663-5)
Supplement: Supplementary file 2 — Additional file 2: Table S1. 146 overlapping DEPs in the CIA/Control group and BZXD/CIA group. [file 12906_2022_3663_MOESM2_ESM.docx]

**Table S1**

146 overlapping DEPs in the CIA/Control group and BZXD/CIA group.

| **28 Day**  **protein ID** | **Gene**  **name** | **Score** | **Unique**  **peptides** | Fc  （CIA/Control） | Fc  (BZXD/CIA） |
| --- | --- | --- | --- | --- | --- |
| B0BN63 | Ahsa1 | 139.56 | 3 | 0.77 | 1.38 |
| B0BNN3 | Car1 | 892.59 | 6 | 1.45 | 0.40 |
| B1WBU9 | Pygm | 137.38 | 9 | 1.32 | 0.71 |
| B2RYI8 | Papss1 | 71.92 | 2 | 0.72 | 1.62 |
| B2RZ77 | Dpt | 836.73 | 8 | 1.21 | 0.60 |
| B5DEX6 | Susd2 | 42.56 | 2 | 1.24 | 0.56 |
| D3Z9M5 | Fkbp7 | 51.1 | 3 | 0.77 | 1.92 |
| D3Z9Z0 | Ank1 | 39.84 | 4 | 1.22 | 0.77 |
| D3ZA84 | Tln2 | 641.14 | 9 | 1.21 | 0.70 |
| D3ZDK4 | LOC102552055 | 52.91 | 2 | 1.59 | 0.55 |
| D3ZDK7 | Pgp | 38.96 | 2 | 1.36 | 1.20 |
| D3ZDT1 | Epb41l2 | 100.83 | 11 | 0.79 | 0.82 |
| D3ZFC6 | Itih4 | 1749.04 | 27 | 1.30 | 1.30 |
| D3ZH41 | Ckap4 | 400.44 | 20 | 0.79 | 2.24 |
| D3ZK34 | RGD1565894 | 67 | 2 | 0.82 | 2.26 |
| D3ZM21 | Comtd1 | 50.57 | 2 | 0.80 | 1.58 |
| D3ZMY7 | Nt5c2 | 32.29 | 2 | 0.73 | 1.50 |
| D3ZN64 | Col28a1 | 38.07 | 2 | 1.39 | 0.65 |
| D3ZRM9 | LOC100360491 | 76.22 | 6 | 0.74 | 0.80 |
| D3ZUL3 | Col6a1 | 7196.5 | 22 | 1.22 | 0.51 |
| D3ZVB7 | Ogn | 3586.56 | 13 | 1.41 | 0.60 |
| D3ZVD7 | Kera | 127.19 | 7 | 0.81 | 0.79 |
| D3ZYK8 | Mmp9 | 0 | 2 | 1.30 | 1.83 |
| D4A5G8 | Pdha1l1 | 144.81 | 5 | 0.82 | 0.60 |
| F1LMU0 | Myh4 | 2558.59 | 18 | 1.51 | 0.70 |
| F1LPS6 | Ifit1 | 41.71 | 2 | 0.75 | 1.73 |
| F1LQ00 | Col5a2 | 161.81 | 3 | 1.60 | 0.81 |
| F1LQ93 | Col9a1 | 0 | 5 | 0.68 | 0.83 |
| F1LQQ1 | Me1 | 50.9 | 3 | 0.83 | 0.64 |
| F1LRE2 | Igfals | 0 | 2 | 1.24 | 0.82 |
| F1LZH0 | LOC100912707 | 24.27 | 2 | 1.60 | 1.32 |
| F1M8E9 | Lyz2 | 73.61 | 3 | 1.42 | 2.86 |
| F1M9R3 | Abi3bp | 126.32 | 8 | 1.26 | 0.82 |
| F2Z3S8 | Tnnc2 | 102.85 | 4 | 1.67 | 0.59 |
| G3V6E4 | Acbd3 | 82.86 | 4 | 0.81 | 0.80 |
| G3V7H3 | Cfd | 309.85 | 2 | 1.29 | 0.76 |
| G3V7K2 | Lifr | 55.22 | 5 | 1.46 | 0.65 |
| G3V7K3 | Cp | 1814.63 | 31 | 1.28 | 1.24 |
| G3V7V6 | Retsat | 136.79 | 5 | 0.54 | 0.61 |
| G3V9G4 | Acly | 528.37 | 16 | 0.68 | 0.72 |
| G5AKR6 | Ncln | 0 | 2 | 0.68 | 1.36 |
| G5ALS1 | GW7_03778 | 404.22 | 2 | 0.53 | 0.68 |
| G5ALS8 | GW7_03785 | 575.53 | 7 | 0.58 | 0.74 |
| G5AQI3 | GW7_04371 | 63.59 | 2 | 0.55 | 2.02 |
| G5ATA7 | GW7_13731 | 2093.98 | 2 | 1.21 | 0.70 |
| G5AX66 | GW7_15073 | 568.54 | 4 | 0.55 | 0.72 |
| G5B286 | GW7_11958 | 62.06 | 2 | 1.37 | 1.47 |
| G5B5P2 | GW7_11087 | 310.42 | 2 | 0.67 | 0.77 |
| G5BEP3 | GW7_12821 | 260.43 | 3 | 0.83 | 1.33 |
| G5BHR4 | GW7_12277 | 1564.83 | 3 | 1.36 | 1.21 |
| G5BKM2 | Mfap4 | 260.96 | 3 | 1.20 | 0.54 |
| G5BW26 | Nono | 171.78 | 3 | 0.83 | 1.31 |
| G5CA40 | GW7_07412 | 894.44 | 3 | 1.21 | 0.61 |
| H6X2V7 | crp | 543.02 | 6 | 1.25 | 1.27 |
| O08628 | Pcolce | 88.84 | 7 | 0.78 | 1.43 |
| O70210 | Chad | 52.12 | 5 | 1.21 | 0.80 |
| O70513 | Lgals3bp | 141.04 | 4 | 0.72 | 1.82 |
| P00564 | Ckm | 905.15 | 15 | 1.26 | 0.65 |
| P01048 | Kng1 | 2934.69 | 7 | 1.33 | 1.72 |
| P01946 | Hba1 | 3558.24 | 7 | 1.37 | 0.40 |
| P02091 | Hbb | 10429.06 | 2 | 1.25 | 0.46 |
| P02600 | Myl1 | 155.67 | 7 | 1.40 | 0.52 |
| P02793 | Ftl1 | 999.44 | 9 | 1.40 | 0.78 |
| P03957 | Mmp3 | 38.11 | 5 | 1.31 | 3.88 |
| P04466 | Mylpf | 172.06 | 8 | 1.44 | 0.56 |
| P04638 | Apoa2 | 0 | 2 | 2.37 | 2.26 |
| P05943 | S100a10 | 128.19 | 3 | 1.28 | 0.74 |
| P06866 | Hp | 1059.86 | 17 | 1.22 | 1.59 |
| P06907 | Mpz | 125.77 | 6 | 1.21 | 0.52 |
| P07323 | Eno2 | 1728.82 | 3 | 0.73 | 1.29 |
| P08932 | Kng1l1 | 1629.17 | 8 | 1.44 | 1.55 |
| P08934 | Kng2 | 406.34 | 8 | 1.26 | 1.30 |
| P09650 | Mcpt1l1 | 422.79 | 10 | 1.48 | 0.30 |
| P10252 | Cd48 | 64.51 | 2 | 1.32 | 1.21 |
| P11884 | Aldh2 | 1019.2 | 3 | 1.25 | 0.75 |
| P12785 | Fasn | 3799.06 | 68 | 0.63 | 0.55 |
| P14141 | Car3 | 4401.9 | 11 | 1.37 | 0.31 |
| P14272 | Klkb1 | 22.34 | 2 | 1.22 | 1.67 |
| P15865 | Hist1h1d | 774.04 | 7 | 0.79 | 0.63 |
| P16975 | Sparc | 166.23 | 6 | 0.73 | 1.27 |
| P17988 | Sult1a1 | 0 | 2 | 0.74 | 0.41 |
| P18588 | Mx1 | 203.47 | 2 | 0.54 | 1.87 |
| P20059 | Hpx | 3082.23 | 26 | 1.35 | 1.23 |
| P20761 | Igh-6 | 1221.54 | 7 | 1.34 | 2.02 |
| P20762 | LOC362795 | 318.86 | 10 | 1.28 | 1.70 |
| P21961 | Cpa3 | 324.22 | 13 | 1.23 | 0.55 |
| P23562 | Slc4a1 | 385.48 | 16 | 1.42 | 0.42 |
| P27139 | Car2 | 252.67 | 9 | 1.49 | 0.35 |
| P27926 | Hk3 | 55.01 | 2 | 1.23 | 1.43 |
| P28037 | Aldh1l1 | 0 | 3 | 0.78 | 0.78 |
| P29266 | Hibadh | 69.08 | 3 | 0.70 | 0.71 |
| P31211 | Serpina6 | 191.98 | 10 | 1.38 | 0.68 |
| P35704 | Prdx2 | 548.95 | 6 | 1.34 | 0.63 |
| P36201 | Crip2 | 42.65 | 3 | 0.70 | 1.31 |
| P40241 | Cd9 | 49.68 | 4 | 1.23 | 0.58 |
| P41350 | Cav1 | 135.66 | 6 | 1.32 | 0.56 |
| P47853 | Bgn | 1416.02 | 14 | 0.69 | 1.78 |
| P50115 | S100a8 | 265.54 | 3 | 1.30 | 3.06 |
| P50116 | S100a9 | 378.22 | 4 | 1.50 | 3.62 |
| P50339 | Cma1 | 167.52 | 4 | 1.33 | 0.40 |
| P51886 | Lum | 3911.62 | 12 | 1.37 | 0.79 |
| P52873 | Pc | 849.11 | 22 | 0.82 | 0.52 |
| P68370 | Tuba1a | 3363.79 | 3 | 0.74 | 1.47 |
| P70490 | Mfge8 | 31.47 | 9 | 1.23 | 0.59 |
| P97544 | Plpp3 | 48.01 | 2 | 0.81 | 0.66 |
| P97849 | Slc27a1 | 53.5 | 6 | 0.78 | 0.82 |
| Q01129 | Dcn | 2352.43 | 15 | 1.27 | 0.57 |
| Q10758 | Krt8 | 103.1 | 2 | 0.51 | 0.51 |
| Q2Q0I9 | Fndc1 | 64.82 | 2 | 1.44 | 0.76 |
| Q32PX2 | Aimp2 | 26.49 | 3 | 0.80 | 1.38 |
| Q3B8Q2 | Eif4a3 | 29.88 | 3 | 0.76 | 1.36 |
| Q4KM66 | LOC500183 | 3021.91 | 4 | 1.33 | 1.39 |
| Q5EAJ6 | Ikbip | 58.67 | 2 | 0.78 | 1.35 |
| Q5PPN5 | Tppp3 | 161.83 | 3 | 1.25 | 0.60 |
| Q5PQV0 | Ggt5 | 115.22 | 4 | 1.29 | 0.60 |
| Q5RJR9 | Serpinh1 | 3349.83 | 18 | 0.80 | 2.01 |
| Q5RKL5 | Steap3 | 50.48 | 6 | 1.25 | 0.82 |
| Q5U206 | Calml3 | 47.15 | 2 | 1.82 | 0.32 |
| Q5U2V1 | Fkbp10 | 80.23 | 7 | 0.77 | 1.89 |
| Q5U2V4 | Plbd1 | 0 | 2 | 1.34 | 2.17 |
| Q5VLR6 | LOC366772 | 351.35 | 6 | 1.23 | 3.37 |
| Q5XI26 | Stat2 | 95.45 | 3 | 0.64 | 1.99 |
| Q5XIE0 | Anp32e | 46.47 | 2 | 1.26 | 0.83 |
| Q5XIH1 | Aspn | 781.11 | 17 | 1.39 | 0.68 |
| Q62975 | Serpina10 | 85.54 | 4 | 1.22 | 1.30 |
| Q63041 | Pzp | 3857.55 | 56 | 1.43 | 1.74 |
| Q63440 | Plp1 | 49.74 | 2 | 0.71 | 1.40 |
| Q63910 | Hba-a1 | 101.75 | 2 | 1.69 | 0.37 |
| Q64578 | Atp2a1 | 357.39 | 8 | 1.39 | 0.68 |
| Q66HI5 | Fth1 | 148.51 | 8 | 1.50 | 0.79 |
| Q6AXR4 | Hexb | 35.14 | 2 | 0.76 | 2.89 |
| Q6AXS5-2 | Serbp1 | 98.86 | 3 | 0.83 | 1.78 |
| Q6AYQ9 | Ppic | 48.2 | 5 | 0.77 | 2.56 |
| Q6IUR5 | Nenf | 41.79 | 2 | 0.82 | 1.24 |
| Q6LDS4 | Sod1 | 576.89 | 5 | 1.21 | 0.57 |
| Q6MG73 | C2 | 99.6 | 2 | 0.69 | 1.47 |
| Q6MG98 | RT1-Da | 164.5 | 3 | 1.27 | 0.79 |
| Q6P6S4 | Sil1 | 40.39 | 2 | 0.72 | 1.29 |
| Q6P6S9 | Entpd5 | 45.76 | 2 | 1.20 | 1.28 |
| Q6PDW8 | Gpx1 | 228.16 | 7 | 1.26 | 0.79 |
| Q8K551 | Actn3 | 633.97 | 3 | 1.40 | 0.61 |
| Q91ZN1 | Coro1a | 203.07 | 9 | 1.27 | 1.69 |
| Q9EPH1 | A1bg | 292.24 | 11 | 2.48 | 0.38 |
| Q9EQP5 | Prelp | 2434.16 | 21 | 1.61 | 0.77 |
| Q9JKB7 | Gda | 2024.35 | 17 | 1.23 | 0.69 |
| Q9WUH4 | Fhl1 | 82.36 | 6 | 1.23 | 0.67 |
