# Supplementary material for: Quantitative proteomic analysis of Bi Zhong Xiao decoction against collagen-induced arthritis rats in the early and late stages
Source: BMC Complement Med Ther. 2022 Jul 13;22:186. doi: 10.1186/s12906-022-03663-5 (PMC9281147; doi:10.1186/s12906-022-03663-5)
Supplement: Supplementary file 3 — Additional file 3: Table S2. 53 overlapping DEPs in the CIA/Control group and BZXD/CIA group. [file 12906_2022_3663_MOESM3_ESM.docx]

**Table S2**

53 overlapping DEPs in the CIA/Control group and BZXD/CIA group.

| **42 Day**  **protein ID** | **Gene**  **name** | **Score** | **Unique**  **peptides** | Fc  （CIA/Control） | Fc  (BZXD/CIA） |
| --- | --- | --- | --- | --- | --- |
| B0BN55 | Urod | 92.87 | 2 | 0.70 | 1.22 |
| B2RYI8 | Papss1 | 71.92 | 2 | 0.67 | 2.77 |
| B5DFB0 | P3h3 | 57.43 | 2 | 0.71 | 1.24 |
| C0SSW8 | EIF2S3Y | 57.04 | 3 | 0.77 | 1.30 |
| D3ZAP9 | Gpd1l | 505.55 | 4 | 0.76 | 1.25 |
| D3ZIE1 | RGD1566369 | 60.20 | 2 | 0.72 | 1.31 |
| D4A5X8 | Ahcyl1 | 102.08 | 2 | 1.40 | 0.67 |
| D4A7L6 | Rpia | 58.59 | 2 | 1.25 | 0.79 |
| F1LMU0 | Myh4 | 2558.59 | 18 | 1.28 | 0.80 |
| F1LRE2 | Igfals | 0.00 | 2 | 0.77 | 1.31 |
| F1LZF4 | Col6a5 | 358.26 | 2 | 3.00 | 0.56 |
| F2Z3S8 | Tnnc2 | 102.85 | 4 | 1.61 | 0.69 |
| G3V6G1 | Jchain | 209.37 | 2 | 1.32 | 0.79 |
| G3V786 | Akr1b8 | 93.62 | 4 | 1.33 | 0.72 |
| G3V8L1 | Pycard | 110.48 | 4 | 1.21 | 0.83 |
| G3V8L7 | Itgam | 49.44 | 2 | 1.22 | 0.74 |
| G5ALS1 | GW7_03778 | 404.22 | 2 | 0.72 | 0.69 |
| G5AV51 | GW7_05254 | 32.44 | 2 | 0.82 | 1.28 |
| G5AX66 | GW7_15073 | 568.54 | 4 | 0.69 | 0.74 |
| G5B0U1 | GW7_13516 | 96.48 | 3 | 1.22 | 0.82 |
| G5B8P1 | GW7_15288 | 25.04 | 3 | 0.78 | 1.32 |
| G5BK68 | GW7_13070 | 0.00 | 2 | 0.77 | 1.26 |
| O08651 | Phgdh | 160.94 | 8 | 0.76 | 1.25 |
| P02600 | Myl1 | 155.67 | 7 | 1.32 | 0.74 |
| P04466 | Mylpf | 172.06 | 8 | 1.34 | 0.74 |
| P05369 | Fdps | 94.91 | 2 | 0.76 | 1.21 |
| P06214 | Alad | 0.00 | 2 | 0.76 | 1.32 |
| P08494 | Mgp | 0.00 | 2 | 0.79 | 1.24 |
| P09006 | Serpina3n | 1268.38 | 17 | 1.63 | 0.74 |
| P12785 | Fasn | 3799.06 | 68 | 0.49 | 1.25 |
| P13941 | Col3a1 | 128.38 | 6 | 1.40 | 0.83 |
| P18588 | Mx1 | 203.47 | 2 | 0.41 | 1.23 |
| P25409 | Gpt | 40.64 | 2 | 0.77 | 1.25 |
| P47967 | Lgals5 | 129.84 | 4 | 0.75 | 1.49 |
| P61765 | Stxbp1 | 35.35 | 6 | 0.82 | 1.22 |
| P62744 | Ap2s1 | 25.17 | 2 | 0.65 | 1.52 |
| P68370 | Tuba1a | 3363.79 | 3 | 0.78 | 1.38 |
| P70490 | Mfge8 | 31.47 | 9 | 0.61 | 1.29 |
| P70584 | Acadsb | 50.39 | 3 | 0.82 | 1.24 |
| Q02765 | Ctss | 81.55 | 3 | 1.58 | 0.74 |
| Q5M9F7 | Actr10 | 48.07 | 2 | 1.41 | 0.77 |
| Q5U2R8 | Mnda | 24.09 | 3 | 1.42 | 0.82 |
| Q62785 | Pdap1 | 38.91 | 2 | 0.71 | 1.37 |
| Q63440 | Plp1 | 49.74 | 2 | 0.68 | 1.64 |
| Q63910 | Hba-a1 | 101.75 | 2 | 1.42 | 0.73 |
| Q66H94 | Fkbp9 | 70.05 | 6 | 0.83 | 1.22 |
| Q68G33 | Gorasp2 | 105.20 | 2 | 0.70 | 1.42 |
| Q6AXS5-2 | Serbp1 | 98.86 | 3 | 0.79 | 1.32 |
| Q6AYG3 | Prune | 43.50 | 2 | 0.80 | 1.34 |
| Q6LDZ3 | Ptprc | 69.79 | 3 | 1.53 | 0.64 |
| Q794F9 | Slc3a2 | 65.97 | 3 | 0.80 | 1.23 |
| Q8K551 | Actn3 | 633.97 | 3 | 1.49 | 0.69 |
| Q95571 | RT1.A(u | 94.26 | 2 | 1.53 | 0.56 |
