# Supplementary material for: Quantitative proteomic analysis of Bi Zhong Xiao decoction against collagen-induced arthritis rats in the early and late stages
Source: BMC Complement Med Ther. 2022 Jul 13;22:186. doi: 10.1186/s12906-022-03663-5 (PMC9281147; doi:10.1186/s12906-022-03663-5)
Supplement: Supplementary file 4 — Additional file 4: Table S3. Top 33 in PPI network ranked by MNC method. [file 12906_2022_3663_MOESM4_ESM.docx]

**Table S3**

Top 33 in PPI network ranked by MNC method

| **Rank** | **Name** | **Score** |
| --- | --- | --- |
| 1 | Acly | 5 |
| 2 | Fasn | 4 |
| 2 | Pc | 4 |
| 4 | Col5a2 | 3 |
| 4 | Col6a5 | 3 |
| 4 | Col6a1 | 3 |
| 4 | Col3a1 | 3 |
| 4 | Ckm | 3 |
| 4 | Actn3 | 3 |
| 4 | Me1 | 3 |
| 11 | Kng2 | 2 |
| 11 | Klkb1 | 2 |
| 11 | Kng1 | 2 |
| 11 | Mylpf | 2 |
| 11 | Fdps | 2 |
| 11 | Pygm | 2 |
| 17 | Rpia | 1 |
| 17 | Papss1 | 1 |
| 17 | Ppap2b | 1 |
| 17 | Nt5c2 | 1 |
| 17 | Gpt | 1 |
| 17 | Cav1 | 1 |
| 17 | Cfd | 1 |
| 17 | C2 | 1 |
| 17 | Urod | 1 |
| 17 | Alad | 1 |
| 17 | Hk3 | 1 |
| 17 | Eno2 | 1 |
| 17 | Aldh2 | 1 |
| 17 | Akr1b8 | 1 |
| 17 | Hexb | 1 |
| 17 | Hibadh | 1 |
| 17 | Acadsb | 1 |
